# Utilisation des antiparasitaires dans la prévention de la toxoplasmose congénitale: revue systématique et méta-analyse

**DOI:** 10.48327/mtsi.v5i4.2025.753

**Published:** 2025-09-15

**Authors:** Richard AMAGBÉGNON, Aretas Babatoundé Nounnagnon TONOUHÉWA, Magalie DAMBRUN, Célia DECHAVANNE, Aurore OGOUYEMI-HOUNTO, Florence MIGOT-NABIAS, Marie-Laure DARDÉ, Aurélien MERCIER, Dorothée KINDÉ-GAZARD

**Affiliations:** 1 Centre hospitalier universitaire de la mère et de lenfant-Lagune (CHU-MEL), 01 BP 107 Cotonou, Bénin; 2 Unité de recherche sur les maladies transmissibles (URMAT), Université dAbomey-Calavi, 01 BP 2009, Cotonou, Bénin; 3 Université Paris Cité, IRD, Inserm, MERIT, 75006 Paris, France; 4 Service de microbiologie du Centre national hospitalier universitaire -Hubert Koutoukou MAGA (CNHU-HKM), Université dAbomey-Calavi (UAC), Faculté des sciences de la santé (FSS), Bénin; 5 Inserm U1094, IRD U270, Univ. Limoges, CHU Limoges, EpiMaCT - Épidémiologie des maladies chroniques en zone tropicale, Institut dépidémiologie et de neurologie tropicale, OmegaHealth, CHU de Limoges, 87025 Limoges, France

**Keywords:** *Toxoplasma gondii,*, Transmission materno-fœtale, Échec thérapeutique, Spiramycine, Pyriméthamine-sulfadiazine, Cotrimoxazole, Pyriméthamine-sulfadoxine, *Toxoplasma gondii*, Maternal-fetal transmission, Treatment failure, Spiramycin, Pyrimethamine-sulfadiazine, Cotrimoxazole, Pyrimethamine-sulfadoxine

## Abstract

**Introduction:**

Dans le cadre de la prévention de l’infection congénitale à *Toxoplasma gondii,* l’utilisation optimale des molécules à activité antiparasitaire telles que la spiramycine et les associations pyriméthamine-sulfadiazine, cotrimoxazole ou pyriméthamine-sulfadoxine en période prénatale demeure un défi majeur pour limiter la transmission materno-fœtale et réduire les effets liés à l’infection congénitale.

**Matériel et méthodes:**

Une revue systématique des études de cohortes, publiées entre 2000 et 2023, a été menée afin d’avoir une perception globale du risque de transmission materno-fœtale chez les femmes enceintes présentant une infection primaire à *Toxoplasma gondii* traitée ou non. Ce risque de transmission materno-fœtale évalué chez les femmes soumises à un régime thérapeutique correspond au taux d’échec thérapeutique.

**Résultats:**

Le risque de transmission materno-foetale global moyen était estimé à 49% (IC 95%, 36-63%) chez les femmes enceintes infectées en cours de grossesse et ne bénéficiant d’aucun traitement à visée anti-toxoplasmique lorsque les femmes étaient catégorisées en fonction des régions de résidence ou en fonction des systèmes de santé disposant d’une stratégie de prise en charge prénatale ou non. Pour les groupes de femmes enceintes traitées par spiramycine ou par la combinaison pyriméthamine-sulfadiazine, les taux d’échec thérapeutique global moyens étaient respectivement de 16% (IC 95%, 7-26%) et de 11% (IC 95%, 3-22%) lorsque les femmes étaient stratifiées en fonction des régions de résidence ou si le système de santé avait une stratégie de dépistage prénatal systématique ou non. L’infection congénitale induit plusieurs atteintes dont la fréquence peut être réduite par la prise en charge de l’infection maternelle. Quant aux régimes thérapeutiques tels que les associations pyriméthamine-sulfadoxine ou sulfaméthoxazole-triméthoprime, les données sont insuffisantes pour une méta-analyse.

**Conclusion:**

Le taux d’échec thérapeutique des anti-toxoplasmiques standards peut être réduit en agissant de façon précoce sur plusieurs facteurs reconnus. La surveillance et l’évaluation des politiques de traitement de l’infection primaire per gravidique doivent être renforcées dans chaque système de santé pour implémenter une stratégie de prévention secondaire mieux adaptée.

## Abstract (en)

**Introduction:**

In the context of preventing congenital *Toxoplasma gondii* infection, optimizing the use of antiparasitic molecules, such as spiramycin, pyrimethamine-sulfadiazine combinations, cotrimoxazole, and pyrimethamine-sulfadoxine, during the prenatal period is a significant challenge in limiting maternal-fetal transmission and reducing associated effects.

**Materials and methods:**

A systematic review of cohort studies published between 2000 and 2023 was conducted to determine the overall risk of mother-to-fetus transmission in pregnant women with primary *Toxoplasma gondii* infection, whether they are treated or not. The risk of maternal-fetal transmission in women undergoing treatment corresponds to the treatment failure rate.

**Results:**

The average overall risk of maternal-fetal transmission was estimated at 49% (95% CI, 36%-63%) among pregnant women infected during pregnancy who did not receive *anti-toxoplasma* treatment. This risk was assessed by categorizing the women according to their region of residence or whether their healthcare system had a prenatal care strategy. Among groups of pregnant women treated with spiramycin or pyrimethamine-sulfadiazine, the average overall treatment failure rates were 16% (95% CI, 7%-26%) and 11% (95% CI, 3%-22%), respectively, when the women were stratified according to region of residence or whether the healthcare system had a routine prénatal screening strategy. Congenital infection can cause several disorders, and the frequency of these disorders can be reduced by treating the maternal infection. There is insufficient data for a meta-analysis of treatment regimens such as pyrimethamine-sulfadoxine or sulfamethoxazole-trimethoprim combinations.

**Conclusion:**

The failure rate of standard *anti-toxoplasma* treatments can be reduced by addressing several recognized risk factors early on. Each healthcare system must strengthen the monitoring and evaluation of treatment policies for primary infection per pregnancy in order to implement a more appropriate secondary prevention strategy.

## Introduction

La toxoplasmose congénitale (TC) est un problème de santé publique encore négligé dans plusieurs pays [30,33]. L’infection congénitale se produit lorsqu’une femme séronégative pour la toxoplasmose contracte l’infection en cours de grossesse. L’incidence de la TC est plus élevée aux États-Unis, en Amérique du Sud et en Afrique en raison de l’absence de prise en charge prénatale contrairement à certains pays européens où il existe un programme de dépistage, d’éducation sanitaire des femmes enceintes séronégatives et de traitement prénatal systématique [9,42]. En l’absence d’une prise en charge optimale, la mort fœtale peut survenir ou les nouveau-nés peuvent présenter des séquelles irréversibles et tardives, notamment de type rétinochoroïdite [24,40]. Les molécules actives vis-à-vis du toxoplasme sont peu nombreuses. Les macrolides sont généralement utilisés en période prénatale dans l’infection maternelle primaire possible ou probable à *Toxoplasma* [34]. Le macrolide le plus fréquemment utilisé est la spiramycine (SPI) qui se concentre dans le placenta pour limiter le passage transplacentaire des tachyzoïtes et prévenir la transmission materno-fœtale [33]. Lorsque l’infection primaire est suspectée par la détection d’anticorps IgM ou IgA chez une femme enceinte séronégative, ou confirmée comme certaine par l’apparition des IgG spécifiques, les inhibiteurs de la synthèse de l’acide folique sont utilisés en période prénatale pour bloquer la transmission materno-foetale. Ils le sont également pour limiter les conséquences liées à l’infection congénitale, laquelle est révélée par la détection d’ADN du toxoplasme dans le liquide amniotique après une amniocentèse recommandée après 18 semaines d’aménorrhée et au moins 4 semaines après l’infection [14,39]. Les antifolates les plus utilisés sont la pyriméthamine et la sulfadiazine (PSZ), peu accessibles dans de nombreux systèmes sanitaires en Afrique. Certains auteurs proposent d’emblée l’usage systématique de l’association pyriméthamine-sulfadiazine dès la découverte de l’infection primaire probable à partir du 2^e^ trimestre de grossesse [9,29]. D’autres antiparasitaires sont peuutilisés à savoir: l’association sulfaméthoxazoletriméthoprime (SMX/TMP) prescrit en traitement prophylactique et l’association pyriméthaminesulfadoxine (PSX) pour le traitement [3,10]. La pyriméthamine-sulfadoxine est généralement accessible dans les pays d’endémie palustre en Afrique subsaharienne comme étant un antiparasitaire recommandé en traitement préventif intermittent du paludisme chez la femme enceinte [22]. Une supplémentation en acide folinique est admise dans le cas d’usage des sulfamides [1,39]. Plusieurs auteurs rapportent un effet bénéfique du traitement précoce et du type de traitement sur le risque de transmission materno-fœtale par rapport aux groupes de femmes qui ne reçoivent aucun traitement [33].

Cependant, des échecs thérapeutiques et cliniques surviennent avec l’utilisation de ces antiparasitaires [15,33]. De récentes études multicentriques européennes montrent que le traitement de l’infection congénitale en période postnatale ne prévient pas l’apparition ou les rechutes des lésions oculaires [17,29]. En l’absence de signes cliniques à la naissance, les enfants congénitalement infectés peuvent, malgré un traitement, développer à long terme des atteintes oculaires récurrentes [18]. Plusieurs facteurs peuvent influencer la survenue de ces récidives. La virulence des souches du parasite, le faible statut socio-économique, un retard dans la détection de l’infection primaire et dans la mise en route du traitement semblent contribuer à une issue défavorable pour le nouveau-né et plus encore pour l’adolescent [18]. Des études antérieures rapportent un taux de rechute des lésions chez les enfants traités qui peut monter jusqu’à 30% pour les cohortes européennes tandis que pour les enfants sud-américains, ce taux peut excéder 70% [18]. Au regard de ces différents aspects, l’utilisation des antiparasitaires en période prénatale demeure un défi capital dans la lutte contre l’infection congénitale afin de limiter la transmission materno-fœtale et les séquelles liées à la toxoplasmose. Il s’avère nécessaire de déterminer quel serait le traitement le mieux adapté chez la femme enceinte pour réduire au maximum le risque de transmission maternofœtale. La présente étude se propose de répondre à cette interrogation au travers d’une revue systématique des études de cohortes publiées entre 2000 et 2023 sur le traitement prénatal de la toxoplasmose chez la femme enceinte.

## Méthodologie

Selon les recommandations du protocole PRISMA [32], nous avons effectué une revue bibliographique afin d’identifier les travaux de recherche ayant porté sur l’efficacité des antiparasitaires utilisés chez la femme enceinte pour le traitement préventif ou curatif de la toxoplasmose congénitale. Les recherches ont été effectuées dans quatre bases de données bibliographiques: Cochrane library, Pubmed, Scopus et Google scholar. Dans chacune de ces bases de données, les mots-clés spécifiés étaient: *(Toxoplasma* OR toxoplasmosis) AND (treatment OR therapy OR drug therapy) AND (pregnant).

Sur la base du titre et du résumé, l’ensemble des travaux de recherche identifiés dans les bases de données *via* les mots clés spécifiés plus haut ont été étudiés et une partie a été incluse dans la synthèse quantitative selon les critères d’éligibilité suivants:

il s’agissait d’une étude de cohorte publiée de 2000 à 2023 même lorsque les données recueillies étaient antérieures, et ayant évalué un antiparasitaire pour prévenir la transmission materno-fœtale de l’infection à *T. gondii*;
le traitement s’était déroulé en période prénatale chez la femme;
l’instauration du traitement prophylactique était subordonnée à une infection maternelle primaire possible ou probable;
l’instauration du traitement curatif en période prénatale était subordonnée à une infection maternelle primaire certaine;
le traitement prophylactique utilisé était la SPI ou le SMX/TMP;
le traitement curatif était constitué de l’une des combinaisons thérapeutiques: (i) PSZ ou (ii) PSX avec une supplémentation en acide folinique. Ces traitements étaient initiés à partir du second trimestre de grossesse en remplacement de la chimioprophylaxie à la SPI lorsque l’infection maternelle primaire était certaine;
le contrôle de l’infection congénitale avait été effectué en période prénatale par la détection de l’ADN de *T. gondii* dans le liquide amniotique ou en période néonatale ou postnatale par la détection des IgM ou IgA antitoxoplasmiques durant les six premiers mois de vie ou la persistance ou l’augmentation des IgG néoformées pendant la première année de vie. La confirmation de l’absence de toxoplasmose congénitale nécessite la constatation d’une sérologie négative vers l’âge d’un an;
le nombre de cas de transmission maternofœtale après traitement chez les femmes enceintes était disponible dans les études.

Par ailleurs, les revues de littérature ou métaanalyses, les études transversales et celles publiées avant l’année 2000 étaient exclues.

Pour chaque étude éligible, les données suivantes ont été extraites dans un tableur Excel: nom du premier auteur, année de publication, pays dans lequel la recherche a été effectuée, taille de l’échantillon, méthode de diagnostic utilisée, traitement préventif ou curatif administré, nombre de femmes infectées pendant la grossesse et soumises au traitement, période d’infection maternelle, nombre de cas d’infections transmises après traitement.

Chaque étude éligible était située par rapport à la région d’étude et à la stratégie de prévention recommandée par le système de santé dans la prévention de l’infection prénatale. Certains systèmes de santé ont adopté une stratégie de dépistage prénatal systématique (DPN) et d’autres n’avaient pas de recommandation nationale (PDR) en matière de prévention de l’infection congénitale. Ces données permettent d’évaluer le risque de transmission materno-fœtale de la toxoplasmose qui rend compte de la capacité de *T. gondii* à infecter le nouveau-né par franchissement de la barrière transplacentaire pendant la grossesse. Ce risque de transmission peut être évalué dans le groupe des infections maternelles non traitées; ce même risque évalué après traitement des cas d’infection maternelle primaire est assimilé au taux d’échec thérapeutique.

L’agrégation des taux de transmission pour les cas d’infection maternelle primaire non traités ou soumis au régime thérapeutique ainsi que la comparaison de leurs efficacités ont été effectuées en utilisant l’approche méta-analytique *via* le logiciel statistique R Software version 3. 8. L’hétérogénéité entre les études a été évaluée en utilisant l’indice d’hétérogénéité I^2^ de Higgins où I^2^<0,25 traduit une hétérogénéité faible, I^2^ compris entre 0,25 et 0,5 indique une hétérogénéité modérée et I^2^>0,5 une hétérogénéité importante [12]. Tenant compte d’une valeur de I^2^ calculée, qui était supérieure à 50%, le modèle à effet aléatoire a été utilisé pour grouper les différents risques de transmission observés pour chaque traitement. Enfin, une analyse des différents taux d’échec thérapeutique en fonction des régimes étudiés a été réalisée par rapport aux variables « région d’étude » ou « stratégie nationale de prévention » par le biais d’une analyse de sous-groupes.

## Résultats

**Figure 1:**
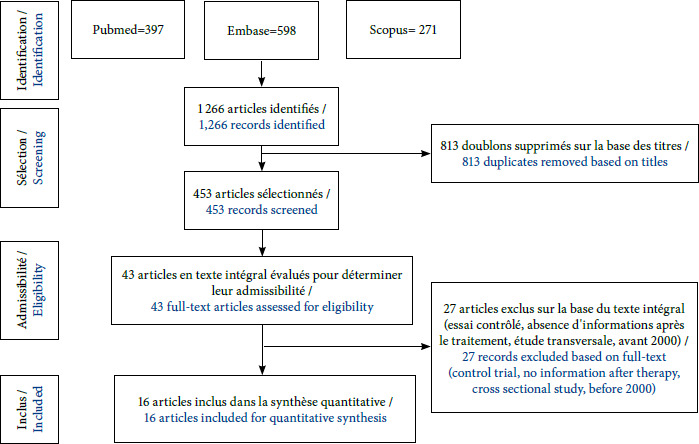
Organigramme de la revue systématique portant sur les schémas thérapeutiques

**Figure 2:**
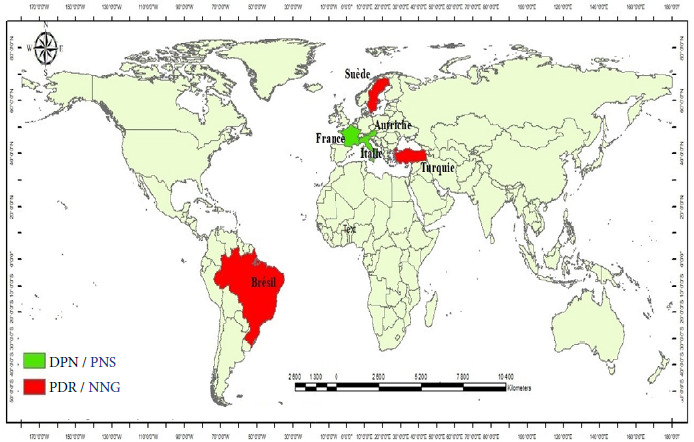
Cartographie des pays éligibles pour l’étude

Au total, 1 266 articles ont été identifiés dans les différentes bases de données bibliographiques. Mais, seules 16 études de cohortes publiées entre 2000 et 2023 étaient éligibles sur la base des critères d’inclusion et d’exclusion préétablis (Fig. 1). Ces études prenaient en compte 4 178 cas documentés d’infection possible, probable ou certaine, contractée pendant la grossesse en Europe, en Amérique du Sud, en Turquie et en Autriche (Fig. 2). Pour ce qui concerne le type de régime thérapeutique, 2 852 femmes enceintes présentant une infection maternelle primaire étaient soumises à la SPI, 385 à l’association PSZ, 80 à l’association PSX, 243 à l’association SMX/ TMP et 618 femmes enceintes n’avaient été soumises à aucun régime thérapeutique (NT). Les différents régimes adoptés sont résumés dans le Tableau I. Pour ce qui concerne le régime SMX/TMP, trois études menées en Italie étaient éligibles. Ces études permettaient de recenser 97,70 et 76 cas d’infection maternelle primaire pour respectivement 8,4 et 2 cas de transmission materno-fœtale post traitement. Pour le régime PSX, deux études réalisées en France étaient éligibles et rapportaient respectivement 60 et 20 cas d’infection maternelle primaire pour 8 cas de transmission materno-fœtale post traitement pour la seconde (Tableau I). Ces données n’étaient pas suffisantes pour entreprendre une vérification de l’homogénéité entre les études afin de mener aisément une méta-analyse. Ce tableau comporte également les données des cas de transmission materno-fœtale avérés chez ces femmes présentant une infection maternelle primaire. Plusieurs tests sérologiques ont été utilisés pour le dépistage de l’infection maternelle en cours de grossesse. Il s’agit notamment des méthodes ELISA *(Enzyme-Linked Immuno Sorbent Assay),* ISAGA *(Immuno-Sorbent Agglutination Assay),* IFAT *(IndirectFluorescent Antibody Test),* CMIA *(Chemiluminescent Microparticle Immuno Assay)* et MEIA *(Microparticle Enzyme Immuno-Assay).* Pour le dépistage de l’infection fœtale, certaines études avaient associé au diagnostic clinique des tests sérologiques de détection des anticorps IgM, IgA ou d’anticorps IgG néoformés chez le nouveau-né. D’autres études avaient établi le diagnostic de l’infection congénitale par l’utilisation de la PCR sur le liquide amniotique après amniocentèse ou sur le sang du cordon ou l’inoculation aux souris.

**Tableau I:** Données des 16 articles retenus pour l’analyse des régimes standard de traitement de la toxoplasmose

Auteurs	Type d’étude	Pays	Stratégie nationale	Année de publication	Région d’étude	Période de collecte des données	Critères d’infection chez les mères*	Nombre d’infections maternelles primaires	Traitement	Nombre de TC**	Période du diagnostic de TC
Gomes Ferrari Strang 2023 [20]	Cohorte	Brésil	PDR	2023	Amérique du Sud	2017-2021	Probable, certaine	28	PSZ	1	Néo et postnatale
								20	SPI	3	
								13	NT	7	
Buonsenso 2022 [10]	Cohorte	Italie	DPN	2022	Europe / Europe	1992-2019	Possible, probable, certaine / Possible probable, certain	97	SMX/TMP	8	Néo et postnatale
								55	SPI	11	
								8	PSZ	1	
Damar Cakirca 2022 [13]	Cohorte	Turquie / Turkey	PDR	2022	Europe-Asie / Europe-Asia	2016-2022	Probable / Probable	87	SPI	1	Néo et postnatale
								13	NT	4	
Bartholo 2020 [6]	Cohorte	Brésil	PDR	2020	Amérique du Sud	2014-2016	Probable, certaine	23	SPI	4	Anté et postnatale / Ante- and postnatal
								3	NT	1	
Avci 2016 [4]	Cohorte	Turquie / Turkey	PDR	2015	Europe-Asie / Europe-Asia	Avant 2015	Probable, certaine	55	SPI	0	Prénatale / Prenatal
								6	NT	4	
Avelino 2014 [5]	Cohorte	Brésil	PDR	2014	Amérique du Sud	2003-2011	Probable, certaine	120	SPI	70	Néo et postnatale
								115	NT	84	
Rodrigues 2014 [38]	Cohorte	Brésil	PDR	2014	Amérique du Sud	2004-2011	Possible, probable	44	SPI	27	Néo et postnatale
								24	NT	19	
Valentini 2015 [44]	Cohorte	Italie	DPN	2015	Europe	1992-2011	Possible Probable, certaine	43	SPI	9	Néo et postnatale
								10	PSZ	1	
								70	SMX/TMP	4	
Fricker- Hildalgo 2013 [16]	Cohorte	France	DPN	2013	Europe	2002-2011	Probable	22	SPI	0	Néo et postnatale
Campello Porto et Duarte 2012 [11]	Cohorte	Brésil	PDR	2012	Amérique du Sud	1994-2009	Probable, certaine	210	SPI	17	Néo et postnatale
								35	PSZ	1	
242	NT	40
Bessières 2009 [7]	Cohorte	France	DPN	2009	Europe	1994-2005	Probable, certaine	60	PSX	0	Néo et postnatale
								216	SPI	66	
Prusa 2015 [36]	Cohorte	Autriche	DPN	2015	Europe	1992-2008	Probable	1 007	SPI	87	Néo et postnatale
								63	NT	32	
Valentini 2009 [43]	Cohorte	Italie	DPN	2009	Europe	Avant 2009	Possible	76	SMX/TMP	2	Néo et postnatale
								74	SPI	0	
Mazzola 2007 [31]	Cohorte	Italie	DPN	2007	Europe	1999-2004	Probable, certaine	47	SPI	8	Anténatale
								2	PSZ	0	
								9	NT	3	
Gilbert 2003 [19]	Cohorte	France, Italie	DPN	2003	Europe	1996-2000	Possible, probable, certaine	800	SPI	82	Néo-postnatale
								195	PSZ	55	
								94	NT	58	
		Autriche	DPN	2003	Europe	1996-2000	Certaine	107	PSZ	2555	
		Suède	PDR	2003	Europe	1996-2000	Possible, probable	12	NT	4	Néo-postnatale
Bessières 2001 [8]	Cohorte	France	DPN	2001	Europe	1986-1996	Possible, probable	20	PSX	8	Néo et postnatale
								29	SPI	14	
								24	NT	11	
**Total**								**4 178**		**772**	

SPI: Spiramycine, PSZ: Pyriméthamine-sulfadiazine, PSX: Pyriméthamine-sulfadoxine

SMX/TMP: Sulfaméthoxazole-triméthoprime

NT: Non traité, DPN: Dépistage prénatal systématique, PDR: Pas de recommandations nationales

* Classification de Lebech et al. [25]

** TC: toxoplasmose congénitale

**Figure 3:**
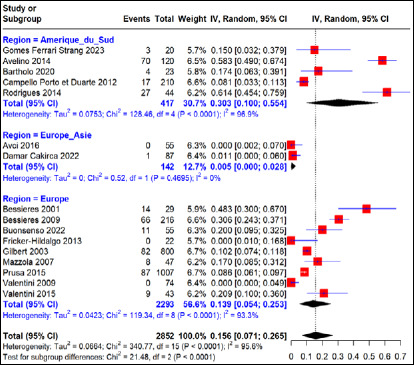
Taux d’échec thérapeutique après traitement à la spiramycine en fonction des régions d’étude

Cette méta-analyse rapporte un taux d’échec thérapeutique global moyen, dans les cas d’infection maternelle primaire traités à la SPI, de 16% (IC 95%, 7-26%) pour 2 852 cas d’infection maternelle primaire, dont 30,7%, 56,6% et 12,7% provenaient respectivement des régions Amérique du Sud (Brésil), Europe (France, Italie, Autriche, Suède) et Europe-Asie (Turquie) avec une hétérogénéité significative entre les régions (I^2^=95,6%, p<0,01) (Fig. 3). Le taux d’échec thérapeutique intra-région était estimé à 30% (IC 95% 10-55%) pour 417 cas d’infection maternelle primaire traités en région Amérique du Sud avec une grande variabilité entre les études menées dans cette région (hétérogénéité I^2^=96,9% (p<0,01). Pour ce qui concerne les études menées en région européenne, un taux d’échec thérapeutique de 14% (IC 95%, 5-25%) était observé pour 2 293 cas d’infection maternelle primaire traités avec une grande variabilité entre les études (I^2^=93,3% p<0,01). Dans la région Europe-Asie et plus précisément en Turquie, cette méta-analyse relate un taux d’échec thérapeutique à la SPI estimé à 0,5% (IC 95%, 0-28%) pour 142 cas d’infection maternelle primaire traités avec une absence d’hétérogénéité entre les études (p>0,05).

**Figure 4:**
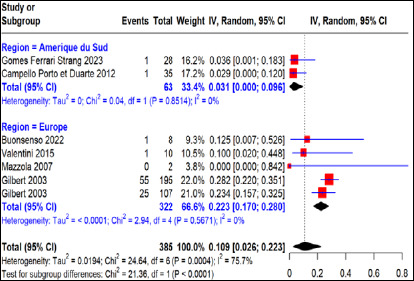
Taux d’échec thérapeutique après traitement à la pyriméthamine-sulfadiazine en fonction des régions d’étude

Le taux d’échec thérapeutique global moyen chez les femmes enceintes soumises au régime PSZ a été estimé à 11% (IC 95% 3-22%) pour 385 cas d’infection maternelle primaire certaine, dont 33,4% et 66,6% provenaient respectivement des régions Amérique du Sud et Europe avec une forte hétérogénéité entre les régions (I^2^=75,7, p<0,01) (Fig. 4). Nos résultats exprimaient un taux d’échec thérapeutique de 3% (IC 95% 0-10%) pour 63 cas d’infection maternelle primaire certaine traités à la PSZ avec une absence de variabilité entre les études menées en Amérique du Sud (I^2^=0%, p>0,05). Pour ce qui concerne les études menées en région européenne, le taux d’échec thérapeutique était de 22% (IC 95% 17-28%) pour 322 cas d’infection maternelle primaire certaine traités à la PSZ avec une variabilité non significative entre les différentes études (I^2^=0%, p>0,05).

**Figure 5:**
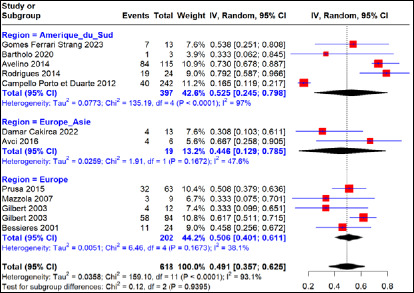
Risque de transmission maternofoetale des cas d’infection maternelle primaire non traités en fonction des régions d’étude

Le risque de transmission materno-foetale global moyen le plus élevé a été observé dans les cas d’infection maternelle primaire non traités. Ce risque de transmission calculé par le modèle à effet aléatoire était de 49% (IC 95% 36-63%) pour 618 cas d’infection maternelle primaire dont 42,6%, 44,2% et 13,2% respectivement dans les régions Amérique du Sud, Europe et Europe-Asie (Turquie), rapportant une grande variabilité entre les études inter-régions (I2=93,1%, p<0,01) (Fig. 5).

Cette variabilité s’est répercutée sur les études intra-régions à travers une hétérogénéité importante en région Amérique du Sud (I^2^=97%, p<0,01) et une hétérogénéité modérée en région européenne (I^2^=38,1%, p>0,05) comme en région Europe-Asie (I^2^=47,6%, p>0,05). Le risque de transmission materno-fœtale était de 52% (IC 95% 24-80%) pour 397 cas d’infection maternelle primaire non traités en région Amérique du Sud, de 51% (IC 95% 40-61%) pour 202 cas d’infection maternelle primaire non traités en région européenne et de 45% (IC 95% 13-78%) pour 19 cas d’infection maternelle primaire non traités en région Europe-Asie.

**Figure 6:**
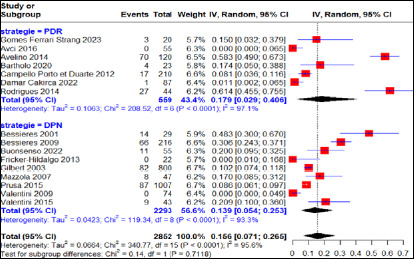
Taux d’échec thérapeutique après traitement à la spiramycine en fonction de la stratégie nationale

La stratification des données en fonction des stratégies relate un taux d’échec thérapeutique global moyen à la SPI de 16% (IC 95%, 7-26%) pour 2 852 cas d’infection maternelle primaire dont 43,4% et 56,6% provenaient respectivement des pays disposant de stratégie PDR et DPN (Fig. 2) avec une grande variabilité entre les stratégies (I^2^=95,6%, p<0,01) (Fig. 6). Pour les systèmes de santé PDR (Brésil, Turquie, Suède), le taux d’échec thérapeutique était de 18% (IC 95% 3-41%) pour 559 cas d’infection maternelle primaire traités à la SPI avec une hétérogénéité significative entres les études (I^2^=97,1% (p<0,01). Pour ce qui concerne les pays disposant stratégie DPN (France, Autriche, Italie), le taux d’échec thérapeutique était de 14% (IC 95%, 4-25%) pour 2 293 cas d’infection maternelle primaire traités à la SPI avec une grande variabilité entre les études (I^2^=93,3%, p<0,01).

**Figure 7:**
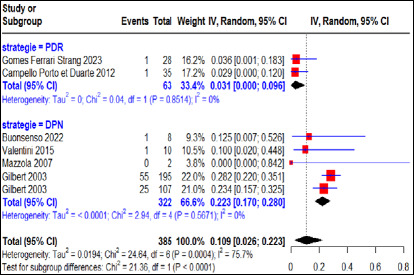
Taux d’échec thérapeutique après traitement à la combinaison pyriméthaminesulfadiazine en fonction de la stratégie nationale

En considérant les femmes enceintes soumises au régime PSZ, le taux d’échec thérapeutique global moyen a été estimé à 11% (IC 95% 3-22%) pour 385 cas d’infection maternelle primaire certaine traités, dont 33,4% et 66,6% proviennent respectivement des pays disposant de stratégie PDR et DPN avec une hétérogénéité importante (I^2^=75,7%, p<0,01) entre les études menées en système PDR ou DPN (Fig. 7). Pour les études menées dans les systèmes de santé de stratégie PDR, le taux d’échec thérapeutique était estimé à

3% (IC 95% 0-10%) pour 63 cas d’infection maternelle primaire certaine traités à la PSZ avec une absence d’hétérogénéité entre les études (I^2^=0%, p>0,05). Dans les systèmes de santé disposant de stratégie DPN, le taux d’échec thérapeutique était de 22% (IC 95% 17-28%) pour 322 cas d’infection maternelle primaire certaine traités à la PSZ avec une absence d’hétérogénéité entre les études (I^2^=0%, p>0,05).

**Figure 8:**
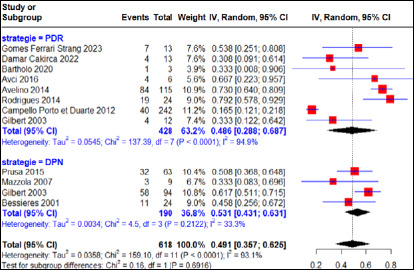
Risque de transmission maternofoetale des cas d’infection maternelle primaire non traités en fonction de la stratégie nationale

Pour les études menées en système de santé disposant de stratégie PDR et DPN, les taux d’échec thérapeutique étaient respectivement de 49% (IC 95% 29-69%) pour 428 cas d’infection maternelle primaire non traités avec une forte hétérogénéité entre les études (I^2^=94,9%, p<0,01) et de 53% (IC 95% 43-63%) pour 190 cas d’infection maternelle primaire non traités avec une hétérogénéité modérée (I^2^=33,3%, p>0,05) (Fig. 8).

## Discussion

Dans cette méta-analyse, nous avons déterminé le risque de transmission verticale de l’infection primaire à *T. gondii* chez les femmes enceintes non traitées et estimé le taux d’échec thérapeutique en fonction des antiparasitaires utilisés. Ces données ont été stratifiées en fonction des régions d’étude et de l’existence ou non de stratégies nationales de dépistage prénatal de l’infection.

Le risque de transmission verticale global moyen en l’absence de prise en charge des cas d’infection primaire à *Toxoplasma* rapporté par notre méta-analyse est de 49% (IC 95% 36-63%) et reste similaire à celui trouvé dans une autre étude méta-analytique publiée en 2021 [50,7% (IC 95% 31,2-70%, p<0,001)] [33]. Ceci traduit la capacité du parasite à infecter le fœtus lorsque la femme est contaminée pendant la grossesse et implique la nécessité d’une prise en charge des cas d’infection maternelle primaire.

La SPI est un antiparasitaire généralement bien toléré qui s’accumule dans le placenta chez la femme enceinte pendant plusieurs semaines et a une activité parasitostatique [27]. Cet antibiotique à activité antiparasitaire utilisé en prophylaxie de l’infection congénitale reste le traitement de référence en première intention de l’infection maternelle primaire à *Toxoplasma* [33]. Dans ce cas, son utilisation révèle un taux d’échec thérapeutique global moyen de 16% (IC 95%, 7-26%) avec une hétérogénéité significative entre les études (I^2^=95,6%, p<0,01) lorsque les données étaient analysées en fonction des régions d’étude ou des stratégies nationales. Ce taux d’échec reste inférieur à ceux de 19,5% (IC 95%, 14-25,5%, p<0,001) et de 30% (p>0,05) que rapportent respectivement une étude méta-analytique regroupant 33 études (32 cohortes et une étude transversale) publiées entre 1974 et 2016 et un essai clinique sur l’efficacité de la SPI [28,33]. Ces différences enregistrées peuvent s’expliquer par plusieurs raisons: i) les divergences dans l’instauration du traitement à la SPI; ii) les données utilisées dans notre méta-analyse sont plus récentes (collectées de 1986 à 2022 et publiées entre 2000 et 2023) et peuvent traduire un début d’optimisation des traitements dans certains systèmes de santé. D’autres auteurs ont rapporté un taux de transmission verticale de 9,9% (IC 95% 5,9%-16,2%) chez les femmes traitées à la SPI seule ou associée à d’autres molécules [45]. Ce taux d’échec thérapeutique à la SPI inférieur à celui obtenu pourrait s’expliquer par le fait que les articles éligibles dans notre méta-analyse ont souffert d’un biais lié à l’absence de distinction entre les cas d’infection primaire possible et les cas d’infection primaire probable, alors que cette clarification pourrait permettre de juger de la fiabilité et de la qualité du diagnostic et faciliter la gestion des cas [25]. Le taux d’échec thérapeutique à la SPI est plus élevé en région Amérique du Sud [30% (IC 95% 10-55)] qu’en région européenne [14% (IC 95% 5-25)] et en Turquie [0,5% (IC 95%, 0-28)]. Ces taux d’échec sont associés à une variabilité élevée (I^2^ > 90% p< 0,01) dans les régions Amérique du Sud et Europe. Le taux d’échec thérapeutique à la SPI élevé en Amérique du Sud peut être lié à un ou plusieurs facteurs: le retard dans d’instauration du traitement; la charge parasitaire élevée; la circulation de souches différentes du type II ou atypiques en Amérique du Sud (ainsi qu’en Afrique) responsables de cas sévères de toxoplasmose chez l’immunocompétent liés à un plus grand tropisme oculaire [21,37]. Le taux d’échec thérapeutique à la SPI en fonction des stratégies était élevé [18% (IC 95% 3-41%)] pour les pays ne disposant pas de stratégie de dépistage prénatal systématique (PDR) par rapport à ceux qui en disposent (DPN) [14% (IC 95%, 4-25%)] et était associé à une hétérogénéité significative entre les études menées selon les deux types de stratégie. Cette situation pouvait s’expliquer par:

l’existence de programmes rigoureux d’éducation primaire et de dépistage prénatal systématique dans les pays de stratégie DPN, traduisant une précocité dans la détection des cas d’infection maternelle primaire ou dans la prise en charge thérapeutique;
une meilleure vigilance des professionnels de santé ou des populations dans les pays de stratégies DPN que dans les pays de stratégie PDR;
la différence dans l’observance du traitement [23,26];
un nombre important de cas d’infection maternelle primaire détectés dans les pays de stratégie DPN, alors que bon nombre de cas semblent être occultés dans les pays de stratégie PDR (56,6% *versus* 43,4% cas d’infection maternelle primaire).

L’effet parasiticide de la combinaison PSZ diminuerait davantage la transmission verticale lorsque le traitement est initié à partir du deuxième trimestre [33]. Dans notre méta-analyse, le taux d’échec thérapeutique global moyen à la PSZ rapporté [11% (IC 95% 3-22%); I2>75,7%, p<0,01] lorsque l’analyse est faite en fonction des régions d’étude ou en fonction des stratégies nationales, était inférieur à celui de 18,5% (p>0,05) rapporté dans un essai clinique multicentrique sur l’efficacité de la PSZ [28]. Ce taux élevé pourrait traduire un retard dans l’instauration du traitement parasiticide. En effet, le traitement commencé dans les 3 semaines suivant la séroconversion réduit la transmission mère-enfant par rapport au traitement commencé après 8 semaines. Il peut aussi être le reflet d’un âge gestationnel plus élevé au moment de la séroconversion associé à un risque accru de transmission materno-fœtale [27,41]. Au regard des données rapportées par région, les taux d’échec thérapeutique à la PSZ plus élevés en Europe [22% (IC 95% 17-28)] qu’en région Amérique du Sud [3% (IC 95% 0-10)] pourraient traduire le fait que la majorité des pays concernés dans les études européennes (excepté la Suède) dispose de stratégie DPN permettant de diagnostiquer plus de cas d’infection maternelle primaire certaine que dans les pays d’Amérique du Sud à stratégie PDR. Le taux d’échec thérapeutique à la PSZ plus élevé dans les régions Europe, Océanie ou dans les pays disposant de stratégie DPN, peut également traduire des cas de séroconversion primaire détectés à un âge gestationnel plus avancé [21]. L’accessibilité de la PSZ étant difficile dans de nombreux pays, il aurait été intéressant de pouvoir évaluer l’efficacité de PSX sur le toxoplasme, d’autant qu’elle possède un effet parasiticide sur le toxoplasme et est utilisée dans plus de 37 pays d’endémie palustre en Afrique subsaharienne en traitement préventif intermittent du paludisme chez la femme enceinte [3,22]. L’utilisation de prises répétées de PSX après le premier trimestre de grossesse, administrée à un mois d’intervalle jusqu’à l’accouchement, est recommandée par l’Organisation mondiale de la Santé et prescrite par les directives du Programme national de lutte contre le paludisme au Bénin [22,35] pour contribuer à la réduction des infections palustres contractées pendant la grossesse. Cette intervention pourrait-elle être un facteur favorisant dans la prévention de la toxoplasmose congénitale? Il faut souligner l’absence d’études africaines dans cette méta-analyse. D’autres molécules telles que l’azithromycine, le cotrimoxazole ou la clindamycine, utilisées par certains cliniciens comme antibiotiques [2,45] et plus facilement accessibles dans de nombreux pays que la PSZ, pourraient permettre d’augmenter la gamme de molécules utilisables. Cependant, l’insuffisance de données n’a pas permis de tirer des conclusions sur leur efficacité dans cette méta-analyse.

## Conclusion

L’amélioration de la prévention de l’infection congénitale à *Toxoplasma* doit résider dans une synergie d’actions entre l’éducation sanitaire, le diagnostic précoce de l’infection primaire périconceptionnelle ou conceptionnelle et la prise en charge thérapeutique optimale. Notre étude montre la réduction du risque de transmission materno-foetale par la mise en place d’un traitement en cas d’infection toxoplasmique, malgré la persistance d’un certain taux d’échec thérapeutique. Le taux d’échec thérapeutique des différents traitements à visée *anti-Toxoplasma* reste difficile à évaluer en l’absence d’algorithme diagnostique et thérapeutique harmonisé dans les différents pays. Des études multicentriques sur différents continents permettraient d’apprécier l’ampleur du niveau de transmission materno-fœtale afin d’implémenter des politiques de prévention de l’infection congénitale mieux adaptées.

## Remerciements

Cette recherche a été menée pour la prévention de l’infection congénitale à *T. gondii* au profit des praticiens et des décideurs et dans le cadre du projet ANR IntroTox (17-CE35-0004). Nous tenons à remercier tous les chercheurs qui y ont contribué pour le bien-être des populations.

## Financement de l’étude

Cette étude n’a reçu aucun financement.

## Contribution des auteurs et autrices

Richard AMAGBÉGNON: rédaction du protocole, revue bibliographique, sélection des articles, extraction des données, analyses statistiques, rédaction du manuscrit.

Aretas Babatoundé Nounnagnon TONOUHÉWA, Magalie DAMBRUN, Célia DECHAVANNE: rédaction du protocole, revue bibliographique, sélection des articles, extraction des données, analyses statistiques, lecture et correction du manuscrit.

Aurore OGOUYEMI-HOUNTO, Florence MIGOT-NABIAS, Marie-Laure DARDÉ, Aurélien MERCIER, Dorothée KINDÉ-GAZARD: validation du protocole, relecture et validation de la version finale.

## Conflit d’intérêt

Aucun conflit d’intérêts n’a été déclaré.
