# Supplementary material for: Use of antiparasitic drugs in the prevention of congenital toxoplasmosis: systematic review and meta-analysis
Source: Med Trop Sante Int. 2025 Sep 15;5(4):mtsi.v5i4.2025.753. [Article in French] doi: 10.48327/mtsi.v5i4.2025.753 (PMC12948362; doi:10.48327/mtsi.v5i4.2025.753)
Supplement: Related texts

# Research article: Clinical aspects of dengue fever in the Savanna region of Togo in 2023

## Introduction

Congenital toxoplasmosis (CT) remains a neglected public health problem in several countries [30, 33]. CT occurs when a seronegative woman contracts the infection during pregnancy. CT is more prevalent in the United States, South America, and Africa due to inadequate prenatal care. In contrast, some European countries have screening programs, educate seronegative pregnant women, and provide systematic prenatal treatment [9,42]. Without optimal care, fetal death may occur, or newborns may experience irreversible and delayed sequelae, particularly retinitis-choroiditis [24,40]. Few molecules are active against *Toxoplasma*. Macrolides are generally used during the prenatal period in cases of possible or probable primary maternal infection with *Toxoplasma* [34]. The most commonly used macrolide is spiramycin (SPI), which concentrates in the placenta to limit the transplacental passage of tachyzoites and prevent mother-to-fetus transmission [33]. When primary infection is suspected based on the detection of IgM or IgA antibodies in a seronegative pregnant woman or is confirmed by the appearance of specific IgG antibodies, folic acid synthesis inhibitors are used during the prenatal period to block maternal-fetal transmission. These inhibitors are also used to mitigate the effects of congenital infection, which is indicated by the presence of *Toxoplasma* DNA in the amniotic fluid following amniocentesis. Amniocentesis is recommended after 18 weeks of amenorrhea and at least four weeks after infection [14,39]. The most commonly used antifolates are pyrimethamine and sulfadiazine (PSZ), which are not widely available in many African health systems. Some authors recommend systematically using the pyrimethamine-sulfadiazine combination as soon as a probable primary infection is detected in the second trimester of pregnancy [9,29]. Other antiparasitic drugs are rarely used: sulfamethoxazole-trimethoprim (SMX/TMP), which is prescribed as a prophylactic treatment, and pyrimethamine-sulfadox-ine (PSX), which is used for treatment [3,10]. Pyrimethamine-sulfadoxine is generally available in malaria-endemic countries in sub-Saharan Africa as an antiparasitic recommended for the intermittent preventive treatment of malaria in pregnant women [22]. Folic acid supplementation is permitted when sulfonamides are used [1,39]. Several authors report that early treatment and the type of treatment have a beneficial effect on the risk of mother-to-child transmission compared to groups of women who receive no treatment [33]. However, therapeutic and clinical failures occur with the use of these antiparasitic drugs [15,33]. Recent European multicenter studies show that treating congenital infection in the postnatal period does not prevent the onset or recurrence of eye lesions [17,29]. Congenitally infected children may develop recurrent eye damage in the long term, even with treatment, in the absence of clinical signs at birth [18]. Several factors may influence the occurrence of these recurrences. These factors include the virulence of the parasite strains, low socioeconomic status, and delays in detecting the primary infection and initiating treatment. These factors appear to contribute to an unfavorable outcome for newborns and even more so for adolescents [18]. Previous studies report relapse rates of up to 30% for treated European children, while for South American children, this rate can exceed 70% [18]. Given these findings, using antiparasitic drugs during the prenatal period is crucial to limiting mother-to-fetus transmission and the sequelae associated with toxoplasmosis. It is crucial to identify the most effective treatment for pregnant women to minimize the risk of transmission from mother to fetus. This study aims to address this issue by conducting a systematic review of cohort studies published between 2000 and 2023 regarding the prenatal treatment of toxoplasmosis in pregnant women.

## Methodology

In accordance with the PRISMA protocol recommendations [32], we conducted a literature review to identify studies on the efficacy of antiparasitic drugs used to prevent or treat congenital toxoplasmosis in pregnant women. The search was conducted in four bibliographic databases: The Cochrane Library, PubMed, Scopus, and Google Scholar. The specified keywords used in each database were: *Toxoplasma* OR toxoplasmosis AND treatment OR therapy OR drug therapy AND pregnant.

Based on the titles and abstracts, we reviewed all the research identified in the databases via the specified keywords, and we included some of it in the quantitative synthesis according to the following eligibility criteria:

it was a cohort study published between 2000 and 2023 that evaluated an antiparasitic drug to prevent maternal-fetal transmission of *T. gondii* infection, even if the collected data was older;
treatment took place during the prenatal period in women;
the initiation of prophylactic treatment depended on possible or probable primary maternal infection;
The initiation of curative treatment during the prenatal period was contingent on a confirmed primary maternal infection.
The prophylactic treatment used was SPZ or SMX/TMP;
Curative treatment consisted of one of the following therapeutic combinations: (i) PSZ or (ii) PSX with folinic acid supplementation. These treatments were initiated in the second trimester of pregnancy, replacing SPI chemoprophylaxis when a primary maternal infection was confirmed.
Congenital infection was monitored prenatally by detecting *T. gondii* DNA in the amniotic fluid. During the neonatal or postnatal period, it was monitored by detecting *anti-toxoplasma* immunoglobulin M (IgM) or immunoglobulin A (IgA) during the first six months of life, or by monitoring the persistence or increase of newly formed immunoglobulin G (IgG) during the first year of life. Confirmation of the absence of congenital toxoplasmosis requires a negative serology test around one year of age.
The number of cases of mother-to-fetus transmission after treatment in pregnant women was reported in the studies.

Furthermore, literature reviews, meta-analyses, cross-sectional studies, and studies published before 2000 were excluded.

The following data were extracted from each eligible study and entered into an Excel spreadsheet: name of the first author, year of publication, country in which the research was conducted, sample size, diagnostic method used, preventive or curative treatment administered, number of women infected during pregnancy who underwent treatment, period of maternal infection, number of cases of infection transmitted after treatment.

Each eligible study was categorized by study region and the recommended prevention strategy of the health system for preventing prenatal infection. Some health systems adopted a routine prenatal screening strategy (PNS), while others had no national guidelines (NNG) for preventing congenital infection. These data allow us to evaluate the risk of maternal-fetal transmission of toxoplasmosis, reflecting the ability of *T. gondii* to infect newborns by crossing the transplacental barrier during pregnancy. The risk of transmission in the group of untreated maternal infections is equated with the treatment failure rate.

We performed a meta-analysis using the statistical software R (version 3.8) to aggregate transmission rates for cases of primary maternal infection that were either untreated or treated, as well as to compare their effectiveness. Heterogeneity between studies was assessed using the I^2^ statistic, where I^2^ < 0.25 indicates low heterogeneity, H between 0.25 and 0.5 indicates moderate heterogeneity, and I2 > 0.5 indicates high heterogeneity. Since the calculated *I^2^* value was greater than 50%, the random effects model was used to group the different observed transmission risks for each treatment. Finally, a subgroup analysis was performed to examine the relationship between treatment failure rates and the variables “study region” or “national prevention strategy.”

## Results

A total of 1,266 articles were identified across various bibliographic databases. However, only 16 cohort studies published between 2000 and 2023 met the pre-established inclusion and exclusion criteria (Fig. 1). These studies accounted for 4,178 documented cases of possible, probable, or confirmed infections acquired during pregnancy in Europe, South America, Turkey, and Austria (Fig. 2). Regarding the treatment regimen, 2,852 pregnant women with a primary maternal infection received SPI; 385 received PSZ; 80 received PSX; 243 received SMX/TMP; and 618 received no treatment. The different regimens adopted are summarized in Table I. Regarding the SMX/ TMP regimen, three studies conducted in Italy were eligible. These studies identified 97, 70, and 76 cases of primary maternal infection and 8, 4, and 2 cases of post-treatment maternal-fetal transmission, respectively. Two studies conducted in France were eligible for the PSX regimen and reported 60 and 20 cases of primary maternal infection, respectively. The second study reported eight cases of post-treatment mother-to-fetus transmission (Table I). However, these data were insufficient to verify homogeneity between the studies, making it difficult to conduct a meta-analysis. The table also includes data on confirmed cases of maternal-fetal transmission in women with primary maternal infection. Several serological tests were used to screen for maternal infection during pregnancy. These included ELISA (enzyme-linked immunosorbent assay), ISAGA (immuno-sorbent agglutination assay), IFAT (indirect fluorescent antibody test), CMIA (chemiluminescent microparticle immunoassay), and MEIA (microparticle enzyme immunoassay). Some studies have used a combination of clinical diagnosis and serological tests to screen for fetal infection. These tests detect IgM, IgA, or newly formed IgG antibodies in newborns. Other studies have used PCR on amniotic fluid after amniocentesis, cord blood, or inoculation in mice to diagnose congenital infection.

**Figure 1:** Flow chart of a systematic review of therapeutics regimens

**Figure 2:** Map of countries eligible for the study

**Table I:** Data from 16 articles selected for analysis of standard toxoplasmosis treatment regimens

Authors	Type of study	Country	National strategy	Year of publication	Study region	Data collection period	Criteria for infection in mothers*	Number of primary maternal infections	Treatment	Number of CT**	Period of CT diagnosis
Gomes Ferrari Strang 2023 [20]	Cohort	Brazil	NNS	2023	South America	2017-2021	Probable, certain	28	PSZ	1	Neo- and postnatal
								20	SPI	3	
								13	NT	7	
Buonsenso 2022 [10]	Cohort	Italy	PNS	2022	Europe	1992-2019	Possible probable, certain	97	SMX/TMP	8	Neo- and postnatal
								55	SPI	11	
								8	PSZ	1	
Damar Cakirca 2022 [13]	Cohort	Turkey	NNS	2022	Europe-Asia	2016-2022	Probable	87	SPI	1	Neo- and postnatal
								13	NT	4	
Bartholo 2020 [6]	Cohort	Brazil	NNS	2020	South America	2014-2016	Probable, certain	23	SPI	4	Ante- and postnatal
								3	NT	1	
Avci 2016 [4]	Cohort	Turkey	NNS	2015	Europe-Asia	Avant 2015	Probable, certain	55	SPI	0	Prenatal
								6	NT	4	
Avelino 2014 [5]	Cohort	Brazil	NNS	2014	South America	2003-2011	Probable, certain	120	SPI	70	Neo- and postnatal
								115	NT	84	
Rodrigues 2014 [38]	Cohort	Brazil	NNS	2014	South America	2004-2011	Probable, certain	44	SPI	27	Neo- and postnatal
								24	NT	19	
Valentini 2015 [44]	Cohort	Italy	PNS	2015	Europe	1992-2011	Possible probable, certain	43	SPI	9	Neo- and postnatal
								10	PSZ	1	
								70	SMX/TMP	4	
Fricker- Hildalgo 2013 [16]	Cohort	France	DPN	2013	Europe	2002-2011	Probable	22	SPI	0	Néo et postnatale
Campello Porto et Duarte 2012 [11]	Cohort	Brazil	PDR	2012	South America	1994-2009	Probable, certain	210	SPI	17	Néo et postnatale
								35	PSZ	1	
								242	NT	40	
Bessières 2009 [7]	Cohort	France	DPN	2009	Europe	1994-2005	Probable, certain	60	PSX	0	Néo et postnatale
								216	SPI	66	
Prusa 2015 [36]	Cohort	Austria	DPN	2015	Europe	1992-2008	Probable	1 007	SPI	87	Néo et postnatale
								63	NT	32	
Valentini 2009 [43]	Cohort	Italy	DPN	2009	Europe	Before 2009	Possible	76	SMX/TMP	2	Néo et postnatale
								74	SPI	0	
Mazzola 2007 [31]	Cohort	Italy	DPN	2007	Europe	1999-2004	Probable, certain	47	SPI	8	Anténatale
								2	PSZ	0	
								9	NT	3	
Gilbert 2003 [19]	Cohort	France, Italy	DPN	2003	Europe	1996-2000	Possible pro bable, certain	800	SPI	82	Néo-postnatale
								195	PSZ	55	
								94	NT	58	
		Austria	DPN	2003	Europe	1996-2000	Certain	107	PSZ	2555	
		Sweden	PDR	2003	Europe	1996-2000	Possible probable	12	NT	4	Néo-postnatale
Bessières 2001 [8]	Cohort	France	DPN	2001	Europe	1986-1996	Possible probable	20	PSX	8	Néo et postnatale
								29	SPI	14	
								24	NT	11	
**Total**								**4 178**		**772**	

SPI: Spiramycin, PSZ: Pyrimethamine-sulfadiazine, PSX: Pyrimethamine-sulfadoxine

SMX/TMP: Sulfamethoxazole-trimethoprim

NT: Not treated, PNS: Routine prenatal screening, NNR: No national guidelines

* Classification by Lebech et al. [25]

** CT: congenital toxoplasmosis

This meta-analysis reports an overall average treatment failure rate of 16% (95% CI, 7-26%) for 2,852 cases of primary maternal infection. Of these cases, 30.7% were from South America (Brazil), 56.6% were from Europe (France, Italy, Austria, and Sweden), and 12.7% were from Europe-Asia (Turkey). There was significant heterogeneity between regions (I^2^ = 95.6%, p<0.01) (Fig. 3). The intra-regional treatment failure rate was estimated at 30% (95% CI, 10%-55%) for 417 cases of primary maternal infection treated in South America. There was considerable variability between studies conducted in this region (I2 = 96.9%, p<0.01). Regarding studies conducted in Europe, a treatment failure rate of 14% (95% CI, 5%-25%) was observed for 2,293 cases of primary maternal infection, with considerable variability between studies (R = 93.3%, p<0.01). In the Europe-Asia region, specifically Turkey, the meta-analysis reported an estimated SPI treatment failure rate of 0.5% (95% CI, 0-28%) for 142 cases of primary maternal infection, with no heterogeneity between studies (p>0.05).

**Figure 3:** Therapeutic failure rate after spiramycin treatment, by study region

The overall mean treatment failure rate for pregnant women receiving PSZ was estimated at 11% (95% CI, 3-22%) for 385 cases of confirmed primary maternal infection. Of these cases, 33.4% were from South America, and 66.6% were from Europe. There was high heterogeneity between regions (I^2^ = 75.7%, p<0.01) (Fig. 4). Our results showed a treatment failure rate of 3% (95% CI, 0%-10%) for 63 cases of confirmed primary maternal infection treated with PSZ. There was no variability between studies conducted in South America (I^2^ = 0%, p>0.05). Regarding studies conducted in Europe, the treatment failure rate was 22% (95% CI, 17%-28%) for 322 cases of confirmed primary maternal infection treated with PSZ. There was no significant variability between the different studies (I^2^ = 0%, p>0.05).

**Figure 4:** Therapeutic failure rate after pyrimethamine-sulfadiazine treatment, by study region

The highest overall average risk of mother-to-fetus transmission was observed in cases of untreated primary maternal infection. Using the random effects model, the calculated risk of transmission was 49% (95% CI: 36%-63%) for 618 cases of primary maternal infection. Of these cases, 42.6%, 44.2%, and 13.2% occurred in South America, Europe, and the Europe-Asia region (Turkey), respectively. There was high variability between interregional studies (I^2^ = 93.1%, p<0.01) (Fig. 5). This variability was reflected in intraregional studies through significant heterogeneity in South America (I^2^ = 97%, p<0.01) and moderate heterogeneity in the European (I^2^ = 38.1%, p>0.05) and Europe-Asia (I^2^ = 47.6%, p>0.05) regions. The risk of mother-to-fetus transmission was 52% (95% CI: 24%-80%) for 397 cases of untreated primary maternal infection in South America, 51% (95% CI: 40%-61%) for 202 cases in Europe, and 45% (95% CI: 13%-78%) for 19 cases in Europe-Asia.

**Figure 5:** Risk of maternal-fetal transmission in untreated cases of primary maternal infection, by study region

Stratifying the data by strategy revealed an average treatment failure rate of 16% (95% CI, 7-26%) for 2,852 cases of primary maternal infection. Of these cases, 43.4% and 56.6% occurred in countries with NNG and PNS strategies, respectively (Fig. 2). However, there was significant variability between strategies (I^2^ = 95.6%, p<0.01) (Fig. 6). For NNG health systems (Brazil, Turkey, and Sweden), the treatment failure rate was 18% (95% CI, 3%-41%) for 559 cases of primary maternal infection treated with SPI. There was significant heterogeneity between studies (I^2^ = 97.1%, p<0.01). In countries with a PNS strategy (France, Austria, and Italy), the treatment failure rate was 14% (95% CI, 4%-25%) for 2,293 cases of primary maternal infection treated with SPI. There was high variability between studies (I^2^ = 93.3%, p<0.01). For pregnant women undergoing PSZ treatment, the overall average treatment failure rate was estimated at 11% (95% CI: 3%-22%) for 385 cases of confirmed primary maternal infection. Of these cases, 33.4% and 66.6% originated from countries with NNG and PNS strategies, respectively. However, there was significant heterogeneity (I^2^ = 75.7%, p<0.01) between studies conducted in NNG or PNS systems (Fig. 7). For studies conducted in health systems with NNG strategy, the estimated treatment failure rate was 3% (95% CI 0-10%) for 63 cases of confirmed primary maternal infection treated with PSZ. There was no heterogeneity between these studies (I^2^ = 0%, p>0.05). In health systems with PNS strategy, the treatment failure rate was 22% (95% CI: 17%-28%) for 322 cases of confirmed primary maternal infection treated with PSZ; again, there was no heterogeneity between studies (I^2^ = 0%, p>0.05). For studies in health systems employing both NNG and PNS strategies, the treatment failure rates were 49% (95% CI: 29%-69%) for 428 untreated cases of primary maternal infection, with high heterogeneity between studies (I^2^ = 94.9%, p<0.01), and 53% (95% CI: 43%-63%) for 190 cases of untreated primary maternal infection, with moderate heterogeneity (I^2^ = 33.3%, p>0.05) (Fig. 8).

**Figure 6:** Therapeutic failure rate after spiramycin treatment according to national strategy

**Figure 7:** Therapeutic failure rate after pyrimethamine-sulfadiazine treatment according to national strategy

**Figure 8:** Risk of maternal-fetal transmission of untreated cases of primary maternal infection according to national strategy

## Discussion

This meta-analysis determined the risk of vertical transmission of primary *T. gondii* infection in untreated pregnant women and estimated the treatment failure rate based on the antiparasitic drugs administered. We stratified these data according to study regions and the presence or absence of national prenatal screening strategies for the infection.

Our meta-analysis found that the overall average risk of vertical transmission in the absence of primary *Toxoplasma* infection management is 49% (95% CI 36%-63%), which is similar to the risk found in another meta-analysis published in 2021 (50.7%, 95% CI 31.2%-70%, p<0.001) [33]. These results reflect the parasite’s ability to infect the fetus when the woman becomes infected during pregnancy, which underscores the importance of managing cases of primary maternal infection. SPI is an antiparasitic drug that is generally well-tolerated and accumulates in the placenta of pregnant women over several weeks. It has parasitostatic activity [27]. This antibiotic, which has antiparasitic properties, is used to prevent congenital infection and remains the first-line treatment for primary maternal infection with *Toxoplasma* [33]. In this case, the overall average treatment failure rate is 16% (95% CI, 7-26%), but there is significant heterogeneity between studies (I^2^ = 95.6%, p<0.01) when the data are analyzed according to study regions or national strategies. This failure rate is lower than the rates of 19.5% (95% CI, 14-25.5%, p<0.001) and 30% reported, respectively, in a meta-analysis of 33 studies (32 cohorts and one cross-sectional study) published between 1974 and 2016 and in a clinical trial on the efficacy of SPI [28,33]. Several possible explanations exist for these differences. First, there may be differences in the initiation of SPI treatment. Second, the data used in our meta-analysis are more recent (collected from 1986 to 2022 and published between 2000 and 2023) and may reflect the beginning of treatment optimization in certain healthcare systems. Other authors have reported a vertical transmission rate of 9.9% (95% CI: 5.9%-16.2%) in women treated with SPI alone or in combination with other molecules [45]. The rate of therapeutic failure with SPI, lower than that obtained, could be explained by the fact that articles eligible for our meta-analysis were biased by the lack of distinction between possible and probable primary infection cases. This distinction could help assess the reliability and quality of the diagnosis and facilitate case management [25]. The treatment failure rate with SPI is higher in South America (30%, 95% CI: 10-55) than in Europe (14%, 95% CI: 5-25) and Turkey (0.5%, 95% CI: 0-28). These failure rates are associated with high variability (I^2^ > 90%, p<0.01) in South America and Europe. The high rate of treatment failure with SPI in South America may be linked to one or more of the following factors: delayed initiation of treatment, high parasite load, and circulation of different type II or atypical strains in South America (as well as in Africa), which are responsible for severe cases of toxoplasmosis in immunocompetent individuals and are linked to greater ocular tropism [21,37]. The rate of treatment failure with SPI was higher for countries with NNG, (18% [95% CI, 3-41%]) compared to countries with PNS (14% [95% CI, 4-25%]). This difference was associated with significant heterogeneity between studies conducted according to the two types of strategies. This could be explained by the following:

Rigorous primary education and routine prenatal screening programs in the PNS strategy countries reflect the early detection of primary maternal infection cases and therapeutic management.
There is greater vigilance among healthcare professionals and populations in the PNS strategy countries than in the NNG strategy countries.
There are differences in treatment adherence [23,26].
A significant number of cases of primary maternal infection are detected in the PNS strategy countries, while many cases appear to be overlooked in the NNG strategy countries (56.6% versus 43.4% of primary maternal infection cases).

The parasiticidal effect of the PSZ combination reduces vertical transmission further when treatment is initiated in the second trimester or later [33].

In our meta-analysis, the reported overall average treatment failure rate for PSZ [11% (95% CI 3-22%); I2>75.7%, p<0.01] when the analysis was performed according to study regions or national strategies, was lower than the 18.5% (p>0.05) reported in a multicenter clinical trial on the efficacy of PSZ [28]. This high rate may reflect a delay in initiating antiparasitic treatment. Indeed, treatment initiated within three weeks of seroconversion reduces mother-to-child transmission compared to treatment initiated after eight weeks. It may also reflect a higher gestational age at seroconversion, which is associated with an increased risk of maternal-fetal transmission [27,41]. Examining the data by region reveals higher PSZ treatment failure rates in Europe (22% [95% CI 17-28]) than in South America (3% [95% CI 0-10]). This could reflect the fact that most countries included in European studies (except Sweden) have PNS strategies that diagnose more cases of confirmed primary maternal infection than South American countries with NNG strategies do. The higher rate of treatment failure with PSZ in Europe, Oceania, and countries with PNS strategies may also reflect cases of primary seroconversion detected at a more advanced gestational age [21]. Since PSZ is difficult to access in many countries, evaluating the effectiveness of PSX on *Toxoplasma* would be interesting, especially since PSX has a parasiticidal effect on *Toxoplasma* and is used in more than 37 malaria-endemic countries in sub-Saharan Africa for intermittent preventive treatment of malaria in pregnant women [3,22]. The World Health Organization and the National Malaria Control Program in Benin [22,35] recommend the use of repeated doses of PSX after the first trimester of pregnancy, administered at one-month intervals until delivery to help reduce malaria infections contracted during pregnancy. Could this intervention contribute to the prevention of congenital toxoplasmosis? It should be noted that this meta-analysis does not include any African studies. Other molecules, such as azithromycin, cotrimoxazole, and clindamycin, which are used by some clinicians as antibiotics [2,45] and are more readily available than PSZ in many countries, could increase the range of molecules that can be used. However, insufficient data prevented conclusions from being drawn about their effectiveness in this meta-analysis.

## Conclusion

Improvements in preventing congenital toxoplasmosis must be based on a combination of health education, early diagnosis of primary periconceptional or periconceptional infection, and optimal therapeutic management. Despite the persistence of a certain rate of treatment failure, our study shows that the risk of mother-to-fetus transmission can be reduced by implementing treatment for *Toxoplasma* infection. However, the treatment failure rate of various *anti-Toxoplasma* treatments is difficult to assess due to the lack of a harmonized diagnostic and therapeutic algorithm across different countries. Multicenter studies on different continents would allow us to evaluate the extent of maternal-fetal transmission and implement more effective prevention policies.

## Acknowledgments

This research, conducted as part of the ANR IntroTox project (17-CE35-0004), aims to prevent congenital *T. gondii* infection and benefit practitioners and decision-makers. We thank all the researchers who contributed to this project for the well-being of the population.

## Funding

This study received no funding.

## Contribution of the authors

Richard AMAGBÉGNON: Protocol drafting, literature review, article selection, data extraction, statistical analysis, and manuscript writing. Aretas Babatoundé Nounnagnon TONOUHÈWA, Magalie DAMBRUN, Célia DECHAVANNE: Protocol drafting, literature review, article selection, data extraction, statistical analysis, and manuscript reading and correction.

Aurore OGOUYEMI-HOUNTON, Florence MIGOT-NABIAS, Marie-Laure DARDÉ, Aurélien MERCIER, and Dorothée KINDÉ-GAZARD contributed to protocol validation and proofreading of the final version.

## Conflict of interest

The authors declare no conflicts of interest.
